# Lost giants, lost functions: palaeodietary insights into the ecological niches of Pleistocene ground sloths

**DOI:** 10.1098/rsbl.2025.0158

**Published:** 2025-10-22

**Authors:** Aditya Kurre, Larisa R. G. DeSantis

**Affiliations:** ^1^ Department of Biological Sciences, Vanderbilt University, Nashville, TN, USA; ^2^ Department of Earth and Environmental Sciences, Vanderbilt University, Nashville, TN, USA; ^3^ Evolutionary Studies, Vanderbilt University, Nashville, TN 37235, USA; ^4^ La Brea Tar Pits and Museum, Los Angeles, CA 90036, USA

**Keywords:** Cenozoic, dental microwear, palaeoecology, Rancho La Brea, stable isotopes, Xenarthra

## Abstract

Ground sloths were terrestrial megafauna that inhabited the Western Hemisphere. While they are inferred to have been browsers and grazers based on craniodental morphology, it is plausible that they performed a wide range of ecological functions, including seed dispersal, bioturbation and nutrient cycling. Understanding ground sloth ecology is challenging due to their enamel-free dentition, which poses limitations to palaeodietary methods, like stable isotope analysis, due to the increased probability of diagenesis in more porous tissues. Here, we conduct dental microwear texture analysis on *Paramylodon harlani* and *Nothrotheriops shastensis* specimens from the La Brea Tar Pits in southern California to compare these species to each other, to co-occurring megafauna and to modern analogues to clarify ground sloth dietary ecology. DMTA of *P. harlani* (i.e. low anisotropy and high complexity) and *N. shastensis* (i.e. low anisotropy and low complexity) suggests that *P. harlani* consumed significantly harder foods (e.g. tubers, roots, seeds, fruit pits) than *N. shastensis*. Findings underscore that these species were not functional replicates of each other or of co-occurring browsers and grazers (e.g. camels and bison). Considering the high degree of dietary overlap in extant folivorous sloths, the extinction of giant ground sloths represents a true loss of ecological function.

## 1. Introduction

Ground sloths (Xenarthra; Folivora) were a group of terrestrial mammals that spread from South to Central and North America during the Great American Biotic Interchange of the late Cenozoic, approximately 3 million years ago [1]. The tiny *Neocnus*, which weighed only approximately 3–4 kg and was adapted to the Caribbean’s dry forests and montane habitats [2], would have been dwarfed by the massive *Megalonyx* (approx. 1000 kg), the most widespread North American species, which ranged as far north as the Yukon Territory in Canada [3]. Still, the largest ground sloth on record is *Eremotherium*, a 6500 kg goliath that roamed South America’s tropical forests and savannas [4] and subsequently migrated throughout Central America and along the gulf coast of North America [5]. Though sloths once exhibited significant diversity, all extant species belong to just two genera: *Choloepus*, two-toed sloths (two species), and *Bradypus*, three-toed sloths (four species). Both groups are restricted to arboreal habitats of the South and Central American neotropics and share common dietary and locomotive behaviours [6,7]. Yet, phylogenetic analyses based on molecular [8–12] and morphological [13–15] data have long depicted that these modern genera emerged from separate monophyletic families, with recent mitogenomic data revealing that two-toed sloths evolved from Megatheridae and three-toed sloths from Mylodontidae [16]. Since all extant sloths seem to have convergently evolved an arboreal niche, this begs the question: how functionally diverse were their ancestors, and what is the impact of such a loss in function?

The functional loss associated with megafaunal decline has profound and often cascading effects on ecosystems [17]. For instance, studies on African elephants (*Loxodonta africana*) have demonstrated that megaherbivores can shape their environments by dispersing large seeds over vast distances and recycling nutrients through dung deposition [18]. Similar studies conducted on extinct North American proboscideans suggest that these megafauna transported limiting nutrients across landscapes in ways that smaller herbivores could not replicate [19]. Over time, alterations to biogeochemical cycles due to megafaunal decline could lead to changes in ecosystem composition. Analyses of fungal spores in North American soil, for instance, have demonstrated that megafaunal decline through the Pleistocene caused increased woody plant encroachment, reduced habitat heterogeneity and altered fire regimes [20]. If ground sloths were truly multifunctional ecosystem engineers, their extinction likely led to a similar decline in ecological function.

**Figure 1.**
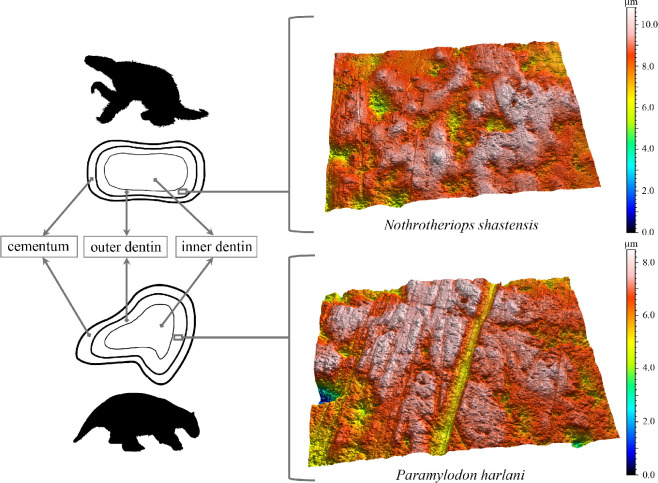
Depiction of ground sloth occlusal surfaces highlighting the area of analysis (outer dentin layer) and representative examples of dental microwear textures from each species examined. Both surfaces display numerous pits and gouges, features indicative of high surface complexity, a metric associated with the consumption of hard materials.

A growing body of research on Pleistocene ground sloth functional diversity has shown that these organisms may have made outsized contributions to their ecosystems. Morphological analyses suggest that different ground sloth species exhibited a wide range of locomotor and craniodental adaptations, allowing for diverse foraging and dietary behaviours that may have contributed to seed dispersal and nutrient redistribution [19,21–26]. Coprolite analyses have provided preliminary insights into the specific plant species that ground sloths consumed [20,27–30]. Additionally, stable isotope analyses of bone collagen have contributed to a better understanding of the relative consumption of C₃ and C₄ vegetation in ground sloths [31–33]. While isotopic results should be interpreted with caution since trophic enrichment factors in ground sloth ecologies are poorly understood, recent work by Tejada and co-authors [34] identified disparate trophic levels occupied by the giant ground sloth genera *Mylodon* and *Nothrotheriops* (via analysis of remarkably preserved hair). Still, only a handful of studies have examined fossilized dentition through either stable isotope analysis of dental tissues or dental microwear analysis [35–37], largely due to the complexities of sloth dentition. Sloths, like other xenarthrans, possess ever-growing teeth made of several layers of dentin (figure 1). Because dentin is more porous than enamel, it is more prone to diagenetic alteration [38], making it less suitable for stable isotope analysis. However, unlike stable isotope analysis, which uses isotopic values within dental tissues as a proxy for diet, dental microwear texture analysis (DMTA) utilizes the microscopic wear features that manifest on dental tissues during mastication—in the short time prior to death—as a dietary proxy [39–49]. DMTA’s reliance on physical, rather than chemical, characteristics of dental tissues means that even the most diagenetically altered dental fossils may be candidates for palaeodietary inference [50]. While the majority of DMTA conducted on vertebrates has focused on enamel [39–44], recent work has established the utility of dentin microwear analysis. In a study by Haupt *et al*. [50], intratooth comparisons of enamel and dentin DMTA attributes in *Puma concolor* revealed that enamel and dentin record certain microwear parameters differently (i.e. *Tfv*, textural fill volume, and *HAsfc_9×9_*, heterogeneity of area-scale fractal complexity using 9 × 9 subdivided grids). However, the study also found that the metrics conventionally used in dental microwear texture interpretation, including *epLsar* (exact proportion Length-scale anisotropy of relief, or anisotropy) and *Asfc* (Area-scale fractal complexity, or complexity), did not vary significantly between dentin and enamel, suggesting that inter-tissue comparisons are possible [50]. Finally, the study found significant differences in dentin microwear textures among extant xenarthran species, specifically revealing that dentin DMTA could differentiate the extant folivorous sloth species *Bradypus variegatus* and *Choloepus hoffmanni* from the opportunist insectivore–omnivore cingulate *Dasypus novemcinctus* [50].

Here, we test hypotheses related to two North American ground sloths: *Paramylodon harlani* and *Nothrotheriops shastensis. Paramylodon* was an approximately 1390 kg sloth that roamed temperate grasslands and woodlands of North America during the late Pleistocene, from Oregon and Texas to central Mexico [51]. We test the hypothesis proposed by Brown (1903) [52] and others that *P. harlani* was primarily a grazer [22,26,29,33,52–54], which predicts high anisotropy and low complexity values due to the dominance of tough grass in the diet. We also test the mixed-feeder hypothesis put forward by Stock [23] and Naples [24] [21,23,24,26,35,36], which anticipates intermediate values for both complexity and anisotropy. Additionally, we examine an auxiliary hypothesis presented by various authors that this species was specifically adapted to excavate and consume subterranean dietary resources [21,23,26], which would likely result in low anisotropy and extremely high complexity values due to increased surface pitting and roughness from a diet of hard plant storage organs, fungi and grit. *N. shastensis*, the smallest North American ground sloth (approx. 460 kg), occupied arid environments of the southwestern USA during the late Pleistocene, with its range extending west to coastal California and south to Belize [51]. Regarding *N. shastensis*, we test the hypothesis that this species consumed desert browse [21,23,25,27,28,30,55], which would produce intermediate anisotropy and complexity values. Lastly, we test the hypothesis that *P. harlani* and *N. shastensis* had indistinguishable dietary ecologies by comparing each species’ DMTA parameters. We then compare each species’ DMTA attribute values to those of their extant relatives (*Bradypus*, *Choloepus*, and more distantly, *Dasypus*) to evaluate the extent of convergence in sloth dietary behaviour over time. All wear facets examined were selected from occlusal surfaces involved in mastication (primarily molars) to ensure functional comparability and avoid confounding wear patterns associated with non-masticatory teeth.

## 2. Material and methods

Ground sloth teeth consist of two orthodentin layers: a softer inner layer and a harder, more mineralized outer layer (figure 1), that is sometimes coated with cementum [56–58]. The inner layer resembles the hardness of ‘dentin’ in other mammals, while the outer layer is harder than typical dentin but softer than enamel (Mohs’ hardness of 3.8 compared to 5.7 of enamel) [50,56,58,59]. In this study, we utilize the terms ‘inner dentin’ and ‘outer dentin’ to avoid confusion [50,59]. All fossil teeth sampled for DMTA are housed in the publicly accessible museum collections at the La Brea Tar Pits and Museum. Dental microwear textures were examined from *N. shastensis* (*n* = 17) and *P. harlani* (*n* = 18) molariform teeth (electronic supplementary material, table S1). Selected specimens featured occlusal surfaces with little to no post-mortem alteration, as indicated by the absence of air pockets, debris or unnaturally smooth surfaces, which typically arise from moulding contamination or inadequate specimen cleaning.

Dental microwear textures were captured using polyvinylsiloxane dental impressions (President’s Jet regular body, Coltène-Whaledent Corp., Cuyahoga Falls, OH, USA) of the occlusal surfaces of the xenarthran teeth. These moulds were then filled with Epotek 301 epoxy resin and hardener (Epoxy Technologies Corp., Billerica, MA, USA) and subsequently left to dry for approx. 36–72 h, to make a clear cast that is optimized for high-magnification microscopy and DMTA. White light confocal profilometry and scale-sensitive fractal analysis were performed on the outer orthodentin layer, which forms the highest reliefs in the taxa studied here and is a functional analogue of enamel [59] (figure 1). Occlusal surfaces were scanned in three dimensions in four spatial quadrants (each 102 × 138 µm^2^) with a 100× objective using a Sensofar PLu neox optical profiler with a 0.73 numerical aperture, white-LED light, sampling resolution of 36.33 data points per 1 µm^2^ and step height of 0.2 µm at the Department of Biological Sciences at Vanderbilt University (see electronic supplementary materials and methods). Scans were processed by SSFA software (Tooth Frax and SFrax, Sufract Corporation), which analyses and quantifies surface attributes such as anisotropy and complexity [40–42]. Mammals with high anisotropy values typically consume tough foods like grass, leaves and/or flesh, while organisms with high complexity values are known to consume hard foods including woody browse, seeds, fruit pits, insects, shelly invertebrates and bone [39–42,44,50,60–64].

**Figure 2.**
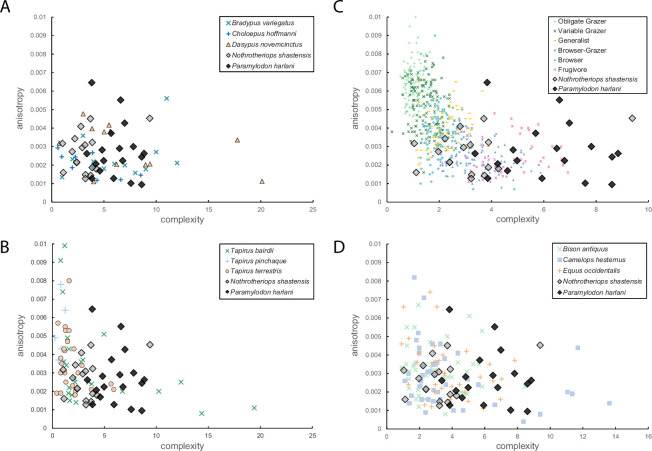
Bivariate plots of complexity vs. anisotropy values comparing extinct ground sloths (*Nothrotheriops shastensis* and *Paramylodon harlani*) against (A) extant xenarthrans (*Bradypus variegatus*, *Choloepus hoffmanni*, *Dasypus novemcinctus*), (B) extant tapirs (*Tapirus bairdii*, *Tapirus pinchaque* and *Tapirus terrestris*), (C) dietary groups of extant bovids (obligate grazer, variable grazer, generalist, browser–grazer and browser, frugivore) and (D) extinct, co-occurring La Brea megaherbivores (*Bison antiquus*, *Camelops hesternus*, *Equus occidentalis*).

Non-parametric statistical tests (Kruskal–Wallis and Dunn’s procedure) were utilized to compare DMTA attribute values of all taxa to account for the non-normal distribution of dental microwear data (Shapiro–Wilk tests, *p* < 0.05). Bonferroni corrections were bypassed to avoid Type II error inflation [65,66]. Cohen’s *d* was calculated to quantify effect size for each comparison, using the conventional thresholds of 0.2 (small), 0.5 (medium) and 0.8 (large) as per Cohen (1988) [67]. Ground sloth data were also compared to extant folivorous and/or omnivorous xenarthrans (figure 2A) [50], folivorous to frugivorous tapirs (figure 2B) [60], co-occurring Rancho La Brea herbivores (figure 2D) [61] and extant bovids that span obligate grazing to frugivorous dietary niches (figure 2C) [44]. Bovid and tapir comparisons are particularly informative since each group exhibits a wide range of dietary habits reflected in their dental microwear textures [44,60]. Although caution must be exercised when comparing DMTA metrics of extinct sloths to extant enamel-bearing taxa due to differences in the way these tooth tissues (enamel and orthodentin) record microwear [50], such comparisons can be informative when focused on attributes that are similarly affected by diet in both tissues. Inter-tissue comparisons therefore focus exclusively on complexity, an attribute typically greatest in organisms that consume hard-food items [39,40,44,50,60–64] and anisotropy, which is highest in organisms that consume tough foods [39,40,44,50,60–64]. All statistical comparisons of *Tfv*, *HAsfc*_3*×3*_ and *HAsfc_9×9_* are noted in electronic supplementary material, table S2.

## 3. Results

All DMTA attribute values are depicted in figure 2, noted in electronic supplementary material, table S1 and summarized in tables 1 and 2 and electronic supplementary material, tables S2–S4.

**Table 1.** Mean microwear values (±s.d., n−1) of *Nothrotheriops shastensis* and *Paramylodon harlani*, compared with values of relevant extant and extinct taxa. Variables include anisotropy (*epLsar*), complexity (*Asfc*) and textural fill volume (*Tfv*).

		***Asfc***		*epLsar*		*Tfv*	
category	taxon/group	mean	s.d.	mean	s.d.	mean	s.d.
extinct ground sloths	*Nothrotheriops shastensis*	3.258	1.833	0.00268	0.00107	7411.897	5654.746
	*Paramylodon harlani*	6.091	1.771	0.00273	0.00149	14 139.104	2454.448
extant xenarthrans	*Bradypus variegatus*	3.783	2.605	0.00243	0.00115	11 165.500	4218.357
	*Choloepus hoffmanni*	3.673	2.607	0.00201	0.00064	12 158.667	4370.150
	*Dasypus novemcinctus*	7.521	5.853	0.00295	0.00123	14 332.583	2058.797
extant tapirs	*Tapirus bairdii*	4.285	4.888	0.00343	0.00238	10 440.917	5368.025
	*Tapirus pinchaque*	0.793	0.302	0.00490	0.00163	7000.714	3806.869
	*Tapirus terrestris*	1.894	1.425	0.00358	0.00158	6021.875	3927.029
extinct megaherbivores	*Bison antiquus*	2.962	1.696	0.00324	0.00129	13 450.541	1548.257
	*Camelops hesternus*	3.963	3.109	0.00287	0.00163	13 250.830	2636.651
	*Equus occidentalis*	3.763	1.830	0.00322	0.00156	13 968.468	1846.719
extant bovid dietary groups	browser-grazer	2.182	0.663	0.00364	0.00136	6531.096	1866.451
	browser	3.510	0.994	0.00226	0.00066	22 534.600	10 8474.626
	frugivore	4.576	1.153	0.00252	0.00103	13 420.879	2130.058
	generalist	2.225	0.651	0.00394	0.00104	9163.732	1793.852
	obligate grazer	1.015	0.263	0.00648	0.00108	2555.158	1081.422
	variable grazer	1.522	0.488	0.00514	0.00131	3736.842	1523.364

**Table 2.** Statistical comparison of microwear variables (*Asfc*, *epLsar* and *Tfv*) between *Nothrotheriops shastensis*, *Paramylodon harlani* and relevant extant and extinct taxa. Bold values indicate significant *p*-values (*α* = 0.05).

		*Asfc*		*epLsar*		*Tfv*	
category	taxon/group	*N. shastensis*	*P. harlani*	*N. shastensis*	*P. harlani*	*N. shastensis*	*P. harlani*
extant xenarthrans	*Bradypus variegatus*	0.401	**0.013**	0.416	0.550	0.137	**0.025**
	*Choloepus hoffmanni*	0.583	**0.013**	0.149	0.210	**0.017**	0.312
	*Dasypus novemcinctus*	**0.003**	0.754	0.633	0.479	**0.000**	0.871
extant tapirs	*Tapirus bairdii*	0.582	**0.001**	0.465	0.433	0.073	**0.021**
	*Tapirus pinchaque*	**0.000**	**<0.0001**	**0.004**	**0.003**	0.802	**0.002**
	*Tapirus terrestris*	**0.013**	**<0.0001**	**0.081**	0.07	0.356	**<0.0001**
extinct megaherbivores	*Camelops hesternus*	0.405	**<0.0001**	0.140	0.095	**<0.0001**	0.125
	*Equus occidentalis*	0.841	**<0.0001**	0.797	0.671	**<0.0001**	0.185
	*Bison antiquus*	**0.398**	**0.001**	0.268	**0.199**	**<0.0001**	0.515
extant bovid dietary groups	obligate grazer	**<0.0001**	**<0.0001**	**<0.0001**	**<0.0001**	**<0.0001**	**<0.0001**
	variable grazer	**<0.0001**	**<0.0001**	**<0.0001**	**<0.0001**	**0.001**	**<0.0001**
	generalist	**0.049**	**<0.0001**	**0.004**	**0.004**	**0.028**	**0.002**
	browser–grazer	**0.026**	**<0.0001**	**0.022**	**0.026**	0.898	**<0.0001**
	browser	0.235	**0.024**	0.292	0.229	**0.000**	0.130
	frugivore	**0.015**	0.466	0.746	0.657	**<0.0001**	0.797

*Paramylodon harlani* exhibits mean complexity values nearly double those of *N. shastensis* (figure 2, table 1). Similarly, textural fill volume is significantly higher in *P. harlani* as compared to *N. shastensis* (table 2). In contrast, anisotropy, *HAsfc_3×3_* and *HAsfc_9×9_* values are indistinguishable (figure 2A, table 2). Notable differences are apparent when comparing extinct taxa to the extant xenarthrans *B. variegatus*, *C. hoffmanni* and *D. novemcinctus*. Specifically, complexity of *P. harlani* is indistinguishable from that of the more omnivorous and insectivorous *D. novemcinctus* but significantly greater than that of both *B. variegatus* and *C. hoffmanni* (figure 2A, tables 1 and 2). In contrast, complexity in *N. shastensis* is indistinguishable from the two folivorous extant sloths (*B. variegatus* and *C. hoffmanni*) and significantly lower than *D. novemcinctus* (figure 2A, table 2). A similar pattern is observed in textural fill volume comparisons, with *P. harlani* being indistinguishable from *D. novemcinctus* and *C. hoffmanni*, but significantly greater than *B. variegatus* (tables 1 and 2). However, *N. shastensis* exhibits significantly lower textural fill volume than *D. novemcinctus*, and *C. hoffmanni*, with indistinguishable values from those of *B. variegatus* (tables 1 and 2). An alternative comparison of extinct sloths using effect size (Cohen’s *d*) reveals large differences in complexity and textural fill volume, with values exceeding 1.61 for both attributes in all comparisons. More specifically, *N. shastensis* exhibits the largest effect sizes (Cohen’s *d* > 1.1) in complexity and textural fill volume when compared to *D. novemcinctus*, while *P. harlani* showed similarly large effect sizes (all Cohen’s *d* > 1.1) relative to both extant sloths’ complexity values (electronic supplementary material, table S4). All other attribute values (anisotropy, *HAsfc_3×3_* and *HAsfc_9×9_*) are indistinguishable between all extant and extinct xenarthran taxa examined (electronic supplementary material, table S2). Because *HAsfc* values fail to account for dietary differences in extant xenarthrans with disparate diets [50], we excluded these attribute values from further discussion (electronic supplementary material, table S2).

*Paramylodon harlani* exhibits significantly greater complexity values compared to all tapirs (figure 2B, tables 1 and 2), while *N. shastensis* has indistinguishable complexity values from *Tapirus bairdii,* but significantly greater values than *Tapirus terrestris* and *Tapirus pinchaque* (figure 2B, tables 1 and 2). When compared to bovids (at the level of dietary category, see S2), *P. harlani* records the highest complexity values, significantly greater than those of obligate grazers, variable grazers, generalists, browser–grazer intermediates and browsers, but indistinguishable from those of frugivores (figure 2C, tables 1 and 2). In contrast, mean complexity values for *N. shastensis* are significantly higher than those of obligate grazers, variable grazers, generalists, browser–grazer intermediates, but indistinguishable from those of browsers (figure 2C, table 1) and significantly lower than those of frugivores (figure 2C, table 2). Interestingly, anisotropy values in both ground sloths are indistinguishable from those of browsers and frugivores but significantly lower than those of all other dietary categories (figure 2C, tables 1 and 2). Differences in DMTA attribute values are observed between ground sloths and co-occurring megaherbivores at Rancho La Brea (figure 2D, tables 1 and 2). Notably, *P. harlani* presents the highest complexity values of all megaherbivores, significantly greater than those of all herbivorous megafauna examined (figure 2D, tables 1 and 2). In contrast, complexity values in *N. shastensis* are indistinguishable from those of all other herbivores including *Bison antiquus*, *Camelops hesternus* and *Equus occidentalis* (figure 2D, table 2).

## 4. Discussion

Ground sloths are hypothesized to have engaged in an extensive breadth of dietary behaviours―from conventional grazing and browsing to rhizophagy and frugivory―each contributing to their unique ecological roles as ecosystem engineers within the Pleistocene [21–31,33,35,36,54,55,68]. Early morphological analysis argued that mylodonts like *P. harlani* were grazers [51–53]. Since then, craniodental analysis of *P. harlani* as well as coprolite analysis of related mylodont species have supported this hypothesis [26,29,33,54,68]. Yet, DMTA data presented here are largely inconsistent with the dietary interpretation that *P. harlani* was a grazer. Notably, anisotropy values (a metric positively correlated with the consumption of tough foods such as grasses) [39–44] of *P. harlani* are significantly lower than those of all grazing taxa analysed and are instead more comparable to frugivores. High complexity values, which are indicative of hard-food consumption, depict that *P. harlani* consumed harder materials than both extant sloths, but similar to *D. novemcinctus* (a species known to forage underground and potentially masticate soil particulates) (figure 2, table 2) [69–72]. Interestingly, a number of extensive palaeoburrow networks found in South America have been attributed to ground sloths, suggesting that some members of this group were capable of extensive digging and subterranean activity [73,74]. While such structures have not been discovered in North America, textural overlap between *P. harlani* and *D. novemcinctus* supports the idea that *P. harlani* may have engaged in similar fossorial behaviours and/or consumed subterranean resources (e.g. roots, tubers, fungus). Additionally, significantly higher complexity values in *P. harlani* as compared to all bovid dietary groups (except frugivores), all extinct megaherbivores and all tapirs (figure 2C, table 2), further point to a highly specialized, hard diet. Several aspects of *Paramylodon* morphology have led researchers to question the ‘grazing hypothesis,’ such as its lack of incisor teeth, its shearing-dependent dental apparatus and its lower relative muzzle width, which would have made grazing an inefficient feeding strategy [21,23,24]; instead, its caniniform teeth, powerful forelimbs with dorsoventrally flattened unguals and high fossorial index would have supported a rhizophagous diet [21,23,24]. Collectively, DMTA findings support the hypothesis that *P. harlani* occupied a highly distinctive dietary niche that may have involved frequent bioturbation. Such regular digging and mastication of plant organs, fungi and other subterranean materials would have been crucial in redistributing nutrients vertically in the soil, influencing plant community dynamics and potentially enhancing soil heterogeneity.

In contrast to the multitude of dietary hypotheses proposed for *P. harlani*, there is consensus that *N. shastensis* was a browser. An array of morphological adaptations in *N. shastensis* support this hypothesis, such as its low relative muzzle width, long tongue and flexible lips to allow for selectivity during browsing [21,23,25]. Coprolite analyses further support that *N. shastensis* was a browser, denoting the consumption of desert browse like yucca (*Yucca* spp.), agave (*Agave* spp.), pine (*Pinus* spp.), mustard (*Brassicaceae*) and saltbush (*Atriplex* spp.) [27,28,30,55]. DMTA data presented here support a browsing dietary hypothesis, with *N. shastensis* anisotropy values indistinguishable from those of extant xenarthran folivores and lower than those of all bovid dietary categories except browsers and frugivores, indicating a lack of tough-food consumption (figure 2A,C, table 2). Additionally, complexity values indistinguishable from those of extant sloths *Choloepus* and *Bradypus* but significantly lower than those of *Dasypus* indicate the consumption of foods with similar hardness as those consumed by extant folivorous xenarthrans, but not as hard as those consumed by burrowing xenarthrans (figure 2A, table 2). Comparisons to bovid dietary groups indicate that *N. shastensis* consumed foods harder than those preferred by obligate grazers, variable grazers, generalists and browser–grazer intermediates, but indistinguishable from those consumed by browsers (and softer than frugivores), while tapir comparisons reveal that *N. shastensis* ingested materials most similar to *T. bairdii*, which is known to consume browse, fruit and large seeds (figure 2B, table 2) [75–77]. Collectively, data from *N. shastensis* corroborate that this species may have played a stabilizing role in shrubland ecosystems, limiting encroachment by woody species and maintaining habitat heterogeneity necessary for other taxa.

Direct comparisons between *P. harlani* and *N. shastensis* indicate that these two ground sloths consumed foods with similar toughness but varying hardness, with complexity and textural fill volume values for *P. harlani* being almost double those of *N. shastensis* (figure 2, table 1). The higher complexity and textural fill volume values for *P. harlani* imply a diet that included harder and/or more mechanically challenging materials like hard-plant components (e.g. plant tubers, seeds, roots) or soil particulates. In contrast, *N. shastensis* may have relied on softer foods and/or engaged in selective browsing. While *P. harlani* and *N. shastensis* overlapped broadly in space and time, they are seldom found at the same locality, leading to the interpretation that ecosystems seldom had the appropriate conditions to support both species at the same time [54,78]. Our data suggest that the coexistence of these species was likely facilitated by the availability of diverse vegetation capable of sustaining their differing dietary needs.

Although modern sloths are commonly viewed as functionally redundant [6,7], the diverse ecological roles of their ancestors demonstrate that ‘sloths’ should not be confined to a single ecological category. As seen with *P. harlani*, ground sloths exhibited diverse diets and filled unique ecological roles often unmatched by co-occurring herbivores. Given ground sloths’ size, dietary flexibility and potential for environmental engineering―such as the dispersal of large-seeded plants and bioturbation—ground sloth extinctions likely had profound, yet understudied, impacts on ecosystem structure and function [17,79–84].

Today, the accelerating pace of human-induced pressures on large herbivores threatens to further erode critical ecological functions [85–89]. Many modern megafauna, such as elephants, bison and tapirs, serve as the last remnants of Pleistocene megaherbivores―yet they too face increasingly severe threats to survival [17]. Recognizing the functional importance of extinct species like giant ground sloths serves as a cautionary tale, emphasizing the urgency of protecting extant megafauna before their irreplaceable ecological roles are lost.

## Data Availability

All new data are provided in the electronic supplementary material, tables included in the manuscript. Further, we reference all data cited. Supplementary material is available online [90].
